# Sense of coherence promotion and occupational and family stress mitigation may improve heart health behaviors in middle-aged working women: a structural equation modelling approach

**DOI:** 10.3389/fpubh.2024.1333867

**Published:** 2024-02-12

**Authors:** Ji Hyun Moon, Eunhye Seo

**Affiliations:** ^1^Department of Community Health Nursing, College of Nursing, Korea University, Seoul, Republic of Korea; ^2^College of Nursing, Keimyung University, Daegu, Republic of Korea

**Keywords:** middle-aged, working women, sense of coherence, psychological stress, health behavior

## Abstract

**Aims:**

This study aimed to construct a model that describes heart health behaviors in middle-aged working women and verify the goodness-of-fit of the model based on Salutogenesis.

**Methods and results:**

This study adopted a cross-sectional design. Participants were 330 middle-aged working women in South Korea. Data were analyzed using structural equation modelling with Sobel’s Z test. In the multiple mediation model, stress coping strategy (*β* = 0.26; *p* < 0.001), social support (*β* = 0.41; *p* < 0.001), and health self-efficacy (*β* = 0.36; *p* < 0.001) had significant direct effects on sense of coherence (SOC). SOC had a significant direct effect on occupational (*β* = −0.72; *p* < 0.001) and family stress (*β* = −0.76; *p* < 0.001). Additionally, SOC (*β* = 0.67; *p* < 0.001), occupational stress (*β* = −0.46; *p* < 0.001), and family stress (*β* = −0.28; *p* < 0.001) had significant direct effects on heart health behaviors. Moreover, SOC had a significantly partial mediating effect on heart health behaviors through occupational stress (*Z* = 3.17; *p* = 0.002) and family stress (*Z* = 2.26; *p* = 0.024).

**Conclusion:**

Occupational and family stress mediated the relationship between SOC and heart health behaviors in middle-aged working women.

**Clinical evidence:**

Interventions that mitigate occupational and family stress may improve heart health behaviors among middle-aged working women.

## 1 Introduction

A recent report (1) from the United States of America underscored the noteworthy trend of increasing women’s labor force engagement, primarily driven by middle-aged women’s increased participation (2). Statistics Korea also suggested this phenomenon, highlighting a consistent and robust 64.8% participation rate among middle-aged women in 2021 (3).

Middle-aged (usually 40–64 years) (4) women experience social, biological, and psychological transitions, that are associated with parenting, parental caregiving, menopausal experiences, and aging-related developmental shifts (5–7). Menopause, a clinical hallmark of middle age, entails hormonal fluctuations that may cause metabolic disorders and develop into cardiovascular disease (8). Indeed, a gender-stratified investigation of cardiovascular disease risk in workers revealed a rapid risk escalation in middle-aged women, underscoring a need to enhance cardiovascular health in this population (9). Nevertheless, middle-aged working women tend to neglect own health management because of the dual challenges of work–life balance (10, 11).

The American Heart Association has recommended strategies to improve modifiable risk factors for cardiovascular diseases, including smoking cessation, adopting a nutritious diet, engaging in physical activity, and maintaining an ideal body mass index (BMI) (12). Considering the impact of middle age in cardiovascular health behaviors on future health status and mortality risk (13), it is essential to promote such behaviors in middle age. For instance, significant changes in hormones, body composition, and lipid profiles during menopause noticeably increase the risk of cardiovascular diseases in middle-aged women (14). However, cardiovascular health behaviors were less widely adopted among those aged 40–59 and 60 and older compared to those aged 20–39 (13). Furthermore, an analysis of the Korean National Health Information Database revealed that approximately 50% of women are engaging in suboptimal exercise levels, which is relatively higher compared to around 38% of men (9, 13).

Excessive and poorly managed psychological stress can make it challenging to adopt heart healthy behaviors (10, 11), which can have adverse effects on cardiovascular health (15). Working women experience work–family conflict and high psychological pressure and stress owing to the difficulties associated with having to conciliate work- and home-life (16). Indeed, the pressure from these multiple roles increases stress and negatively impacts their health (17), ultimately leading to detrimental effects on the cardiovascular health of working women (9).

Middle-aged working women’s heart health behaviors, in the context of workplace and home stressors, can be explained by Antonovsky’s Salutogenesis (18, 19). Salutogenesis pertains to innate resources and internal abilities—which encompass general resistance resources (GRRs) and sense of coherence (SOC)—empowering individuals to overcome stress and uphold well-being despite inevitable stress and disease-related factors (18, 19). GRRs represent deployable assets interlinked with SOC, and the latter reflects one’s ability to understand and manage GRRs and to find meaning from stress and tension states (18, 19). The harmonious interplay of GRRs and SOC facilitates efficacious tension resolution, propelling individuals toward a health-affirming trajectory (18, 19). Considering Salutogenesis, the incapacity of middle-aged working women grappling with stress-induced tension to achieve successful resolution may impede the adoption of heart health behaviors, which relates to cardiovascular health decline.

Factors related to heart health behaviors include stress coping strategy, health self-efficacy, social support, SOC, occupational stress, and family stress (18–24). Specifically, stress coping strategy, social support, and health self-efficacy are known to help individuals adapt to stressful situations, increasing their SOC (18–21), which consequently has a positive effect on occupational stress (22) and family-related stress (23), thus, improving health behaviors (24). Still, these determinants of heart health behaviors (25, 26) have received limited investigations in the context of middle-aged working women.

Thus, this study aimed to establish a comprehensive structural model that describes the direct and indirect influences and provides preliminary indications of potential causal relationships among stress coping strategy, health self-efficacy, social support, SOC, occupational stress, family stress, and heart health behaviors in middle-aged working women. This study delivers reference data for the design of interventions that foster heart health behaviors in middle-aged working women.

### 1.1 Hypothetical model

**Figure 1:**
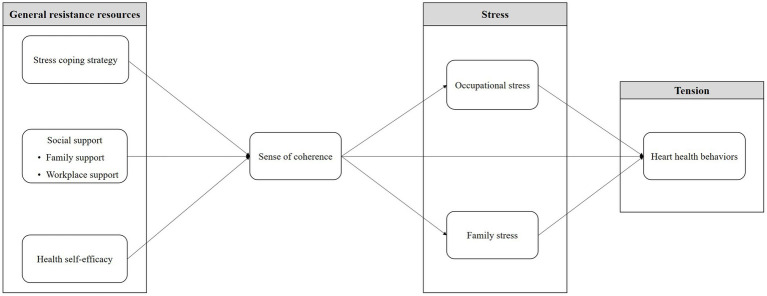
Hypothetical model.

Grounded in the Salutogenesis framework, this research defined stress coping strategy, health self-efficacy, and social support (divided into family support and workplace support) as GRRs (18, 19). Stress was considered to encompass occupational and family stress (16), while heart health behaviors represented health indicators. The model incorporated SOC, a crucial Salutogenesis component (18, 19). Based on our hypothetical model (Figure 1), the hypotheses were as follows: (1) stress coping strategy, health self-efficacy, and social support directly influence SOC (18–21); (2) SOC directly influences occupational and family stress (22, 23) and heart health behaviors (24); (3) SOC indirectly influences heart health behaviors through occupational and family stress; (4) occupational and family stress directly influence heart health behaviors (10, 11).

Considering previous studies based on Salutogenesis (18, 19), stress coping strategy, social support, and health self-efficacy were treated as exogenous variables, while SOC, occupational stress, family stress, and heart health behaviors were endogenous variables. Among the endogenous variables, occupational and family stress factors were defined as mediation factors.

## 2 Methods

### 2.1 Study design and sample

This study adopted a cross-sectional design with a factor analysis, as it enabled us to simplify a set of complex variables using statistical methodologies and to explore the underlying dimensions that explain the relationships among the multiple variables. Furthermore, a total of 330 middle-aged working women from South Korea participated. The inclusion criteria were (1) working women aged 40–64 years, (2) residing in South Korea, and (3) that could be contacted by email. Participants who had any difficulty responding to online surveys were excluded. The study sample comprised 330 participants since Tabachnick, Fidell, and Ullman (27) suggested the need for at least 300 cases, and Comrey and Lee (28) stated that a sample size of 300 was adequate for factor analysis while considering a 10% non-response rate.

### 2.2 Measurements

#### 2.2.1 Sociodemographic and health-related characteristics

Sociodemographic characteristics such as age, education level, occupation, marital status, number of family members, and monthly household income were assessed. Health-related characteristics such as underlying diseases, amount of alcohol consumed (per occasion), smoking status, amount of moderate exercise, weight, and height were also assessed. Moderate exercise was defined as at least 150 min of exercise a week (29). BMI was calculated by dividing the weight (kg) by the square of the height (m^2^), and categorized into ≥25 kg/m^2^ and < 25 kg/m^2^ (30).

#### 2.2.2 Stress coping strategy

Stress coping strategy was assessed using the way of coping scale that was developed by Lazarus and Folkman (31) and modified and translated into Korean by Oh and Han (32). The 33 items are divided into six subscales—problem-focused, wishful thinking, detachment, seeking social support, focusing on the positive, tension reduction—and are measured on a four-point Likert scale. The Cronbach’s α was 0.79 in Oh and Han’s (32) study and 0.82 in this study.

#### 2.2.3 Social support

Social support was investigated from the perspective of family and workplace support; these are the two sources of social support that facilitate and promote heart health behaviors among fulltime working adults (33). Family support was assessed using the standardized 20-item Perceived Social Support–family scale (PSS-Fa) (34). Items concern respondents’ feelings and experiences regarding their family members, where “yes” responses are scored 1 and “no” or “I do not know” responses are scored zero, and total scores range from 0 to 20 points. The Cronbach’s α of this scale was 0.90 in the original study (34) and 0.94 in this study.

Workplace support was measured using the Workplace Support for Health scale (35) to assess participants’ perceptions of the support received in the workplace for a healthy lifestyle. This tool consists of five items measured on a five-point Likert scale. The Cronbach’s α was 0.82 in Kava et al.’s (35) study and 0.93 in this study, showing satisfactory internal consistency in both studies.

#### 2.2.4 Health self-efficacy

Health self-efficacy was measured using the 24-item Korean version of the Self Rated Abilities for Health Practices: Health Self-Efficacy Measure (K-SRAHP) (36), originally developed by Becker et al. (37). It comprises six subscales (i.e., exercise, illness, emotion, nutrition, stress, and health practice) measured on a five-point Likert scale. The Cronbach’s α was 0.91 in the validation study of K-SRAHP (36) and 0.96 in this study.

#### 2.2.5 Sense of coherence

SOC was measured using the 13-item-Short Form Sense of Coherence developed by Antonovsky (19). It comprises three subscales, as follows: comprehensibility (five items), manageability (four items), and meaningfulness (four items). Items are answered on a seven-point Likert-type scale. The Cronbach’s α of this scale ranged from 0.82–0.95 in the original study (19) and from 0.84–0.89 in this study.

#### 2.2.6 Occupational stress

Occupational stress was measured using the 24-item Korean Occupational Stress Scale-short form (KOSS-SF) (38). It encompasses the subscales of job demand, insufficient job control, interpersonal conflict, job insecurity, occupational system, lack of reward, and organizational culture. Items are measured on a four-point Likert scale, and total scores range from 24 to 96 points. The Cronbach’s α of this scale ranged from 0.51–0.85 in the original study (38) and from 0.73–0.89 in this study.

#### 2.2.7 Family stress

Family stress was measured using a 21-item questionnaire developed for married working women by Kim and Cho (39). It comprises the four subscales of cooperation, satisfaction with relationships, democratic and comfortable environment, and disturbance of own living. Answers are measured on a four-point Likert scale. We used 20 items, excluding one item (i.e., I feel the loss of my authority as a parent), because participants who did not have children could not answer. The Cronbach’s α of this scale was 0.86 in the original study (39) and 0.88 in this study.

#### 2.2.8 Heart health behaviors

Heart health behaviors were measured using the 36-item Evaluation Tool for Metabolic Syndrome Modification Lifestyles, originally developed by Kang (40). This scale comprises six subscales: physical activity and weight control, dietary habits, drinking and smoking, sleep and rest, stress, and drug and health management. The items are answered on a four-point Likert-type scale. The Cronbach’s α of the scale was 0.92 in the original study (40) and 0.93 in this study.

### 2.3 Data collection

Participants were recruited through an online survey institute (i.e., Macromill Embrain Co Ltd.) that randomly sent emails to a pool of more than 1.3 million people, and subsequently provided the questionnaire to those who voluntarily responded. All participants were requested to, before agreeing to participate and having their personal information collected, carefully read the explanations regarding the purpose and content of this study, confidentiality, and the right to withdraw. Each participant was given a small incentive of approximately $3 (USD) in accordance with the regulations of the survey institute. The data were collected from October to November 2021.

### 2.4 Statistical analysis

Data were analyzed using SPSS version 23.0 and AMOS version 23.0 for Windows (IBM Corp.). A significance level of *p* < 0.05 was considered for all analyses. Sociodemographic and health-related characteristics and study variables were analyzed using descriptive statistics, including frequency, percentage, mean, and standard deviation. Data normality was analyzed by skewness and kurtosis. Cronbach’s α was computed for the variables. Correlations between study variables were evaluated using Pearson’s correlation coefficients.

A confirmatory factor analysis was performed to validate the hypothesized model. Convergent validity was calculated using factor loading, constructive reliability (CR), and average variance extracted (AVE). A CR value of ≥0.70 and an AVE of ≥0.50 were used for confirming convergent validity (41). Discriminant validity was assessed by correlation coefficient and AVE (ɸ^2^ < AVE) (42).

Structural equation modelling (SEM) with a maximum likelihood estimation method and Sobel’s Z test was used to evaluate the fit of the hypothesized model based on the following multiple criteria: standardized χ^2^ (χ^2^/df) ≤ 3 (43), goodness-of-fit index (GFI) > 0.90, comparative-fit index (CFI) > 0.90, Tucker-Lewis index (TLI) > 0.90, standardized root mean square residual (SRMR) < 0.08, and root mean square error of approximation (RMSEA) < 0.07 (43–45). Hypotheses regarding the structural relationships of the constructs in the final model were evaluated using the magnitude of path coefficients (standardized coefficient) and their significance. The direct, indirect, and total effects of the latent variables were estimated. Sobel’s Z test, a commonly used statistic for testing the significance of mediation effects, was used to determine the indirect effects in the hypothesized model (46).

### 2.5 Ethical considerations

Participants voluntarily agreed to complete the survey, participate in this study, and submitted a written consent form only after receiving and reading recruitment information and research participation instructions. The research protocols were approved by the Institutional Review Board of Korea University (KUIRB-2021-0351-01), and all procedures in this study followed the ethical standards of this board. Furthermore, no animal study was presented in this manuscript, and there are no potentially identifiable images or data are presented in this study.

## 3 Results

### 3.1 Participants’ sociodemographic and health-related characteristics

Table 1 serves merely to characterize participants; we saw no need to control for demographic characteristics in the model, as the mean age of participants was 50.97 years, with a substantial portion of participants (83.6%) in their 40s (40.3%) and 50s (43.3%). Of them, 38.8% were office workers, 74.2% were married, and 47.3% had a monthly household income of 5 million won (47) or more, based on a median-income family of four. Additionally, 9.1% were current smokers, and 17.9% engaged in moderate exercise for 150 min or more per week.

**Table 1:** Participants’ sociodemographic and health-related characteristics (*N* = 330).

Characteristics	N (%) or Mean (SD)
Age (group)	50.97 (6.87)
40 ~ 49	133 (40.3)
50 ~ 59	143 (43.3)
60 ~ 64	54 (16.4)
Education level	
High school graduate	91 (27.6)
College graduate	199 (60.3)
Over graduate	40 (12.1)
Occupation	
Office workers	128 (38.8)
Managers & professionals	70 (21.2)
Sales & service workers	54 (16.4)
Technology	45 (13.6)
Others	33 (10.0)
Marital status	
Married	245 (74.2)
Never married	48 (14.6)
Widowed/separated/divorced	37 (11.2)
Number of family members	
1	23 (7.0)
2	91 (27.6)
3	84 (25.4)
≥ 4	132 (40.0)
Monthly household income (10,000 won)	
≤ 500	174 (52.7)
> 500	156 (47.3)
Underlying diseases	
Cardiovascular disease	6 (1.8)
HTN, DM, Hyperlipidemia	86 (26.1)
Amount of alcohol drinking (glasses)	3.28 (3.19)
Current smoking	
Yes	30 (9.1)
No	300 (90.9)
Amount of moderate exercise (per week)	
≥ 150 min	59 (17.9)
< 150 min	271 (82.1)
BMI (kg/m^2^)	
≥ 25	66 (20.0)
< 25	264 (80.0)

BMI, body mass index; DM, diabetes; HTN, hypertension; SD, standard deviation.

Table 2 shows the descriptions of measured variables. Among the SOC subscales, meaningfulness had the highest mean score (mean = 4.59) and manageability had the lowest mean score (mean = 4.10). Participants had high occupational stress owing to insufficient job control (mean = 2.48). Among the family stress subscales, the mean score for satisfaction with relationships and democratic and comfortable environment was 2.96.

**Table 2:** Descriptive statistics for measured variables.

Latent variables	Measured variables	Mean ± SD	Skewness	Kurtosis
Stress coping strategy	Total	2.66 ± 0.23	−0.16	2.05
	Problem-focused	2.90 ± 0.31	−0.37	1.30
	Wishful thinking	2.61 ± 0.37	−0.45	1.46
	Detachment	2.44 ± 0.40	−0.09	0.35
	Seeking social support	2.66 ± 0.39	−0.52	1.49
	Focusing on the positive	2.83 ± 0.40	−0.49	1.62
	Tension reduction	2.70 ± 0.59	−0.26	0.40
Social support	Family support	1.02 ± 0.08	−0.39	0.08
	Workplace support	3.24 ± 0.83	−0.55	0.33
Health self-efficacy	Total	2.50 ± 0.65	−0.13	−0.35
	Exercise	2.47 ± 0.77	−0.16	−0.33
	Illness	2.55 ± 0.79	−0.36	−0.23
	Emotion	2.43 ± 0.74	−0.04	−0.44
	Nutrition	2.39 ± 0.92	−0.19	−0.64
	Stress	2.35 ± 0.84	−0.39	−0.31
	Health practice	2.86 ± 0.75	−0.30	−0.44
Sense of coherence	Total	4.30 ± 0.90	−0.03	0.26
	Comprehensibility	4.30 ± 1.01	0.05	0.30
	Manageability	4.10 ± 0.94	0.12	0.00
	Meaningfulness	4.59 ± 1.10	−0.14	−0.01
Occupational stress	Total	2.36 ± 0.39	−0.01	0.21
	Job demand	2.34 ± 0.57	0.14	0.12
	Insufficient job control	2.48 ± 0.59	0.04	0.20
	Interpersonal conflict	2.33 ± 0.64	0.33	0.14
	Job insecurity	2.20 ± 0.77	0.18	−0.50
	Occupational system	2.43 ± 0.59	0.51	0.37
	Lack of reward	2.41 ± 0.58	0.53	0.68
	Organizational climate	2.25 ± 0.61	0.15	−0.01
Family stress	Total	2.89 ± 0.42	−0.41	1.20
	Cooperation	2.89 ± 0.49	−0.35	0.87
	Satisfaction with relationships	2.96 ± 0.59	−0.41	0.28
	Democratic and comfortable environment	2.96 ± 0.54	−0.49	1.12
	Disturbance of own living	2.19 ± 0.74	0.31	−0.06
Heart health behaviors	Total	2.55 ± 0.45	0.22	0.19
	Physical activity and weight control	2.14 ± 0.64	0.43	−0.01
	Dietary habits	2.55 ± 0.52	0.13	0.08
	Drinking and smoking	3.46 ± 0.82	−1.65	1.81
	Sleep and rest	2.95 ± 0.62	−0.05	−0.24
	Stress	2.79 ± 0.73	−0.13	−0.36
	Drug and health management	2.50 ± 0.65	0.14	−0.28

SD, standard deviation.

### 3.2 Correlations of study variables

As shown in Table 3, stress coping strategy (*r* = 0.23, *p* < 0.001), health self-efficacy (*r* = 0.49, *p* < 0.001), and social support (*r* = 0.47, *p* < 0.001) were positively correlated with SOC. In contrast, occupational (*r* = −0.53, *p* < 0.001) and family stress (*r* = −0.56, *p* < 0.001) were negatively associated with SOC. These results corroborate the interaction between stress coping strategy, health self-efficacy, social support, and Sense of SOC.

**Table 3:** Correlations of study variables.

Variables	1	2	3	4	5	6	7
	r (*p*)						
1. Stress coping strategy	1						
2. Social support	0.33 (*p* < 0.001)	1					
3. Health self-efficacy	0.36 (*p* < 0.001)	0.47 (*p* < 0.001)	1				
4. Sense of coherence	0.23 (*p* < 0.001)	0.47 (*p* < 0.001)	0.49 (*p* < 0.001)	1			
5. Occupational stress	−0.24 (*p* < 0.001)	−0.54 (*p* < 0.001)	−0.36 (*p* < 0.001)	−0.53 (*p* < 0.001)	1		
6. Family stress	−0.20 (*p* < 0.001)	−0.46 (*p* < 0.001)	−0.43 (*p* < 0.001)	−0.56 (*p* < 0.001)	0.48 (*p* < 0.001)	1	
7. Heart health behaviors	0.32 (*p* < 0.001)	0.43 (*p* < 0.001)	0.67 (*p* < 0.001)	0.44 (*p* < 0.001)	−0.25 (*p* < 0.001)	−0.38 (*p* < 0.001)	1

### 3.3 Validity of measurement model

**Figure 2:**
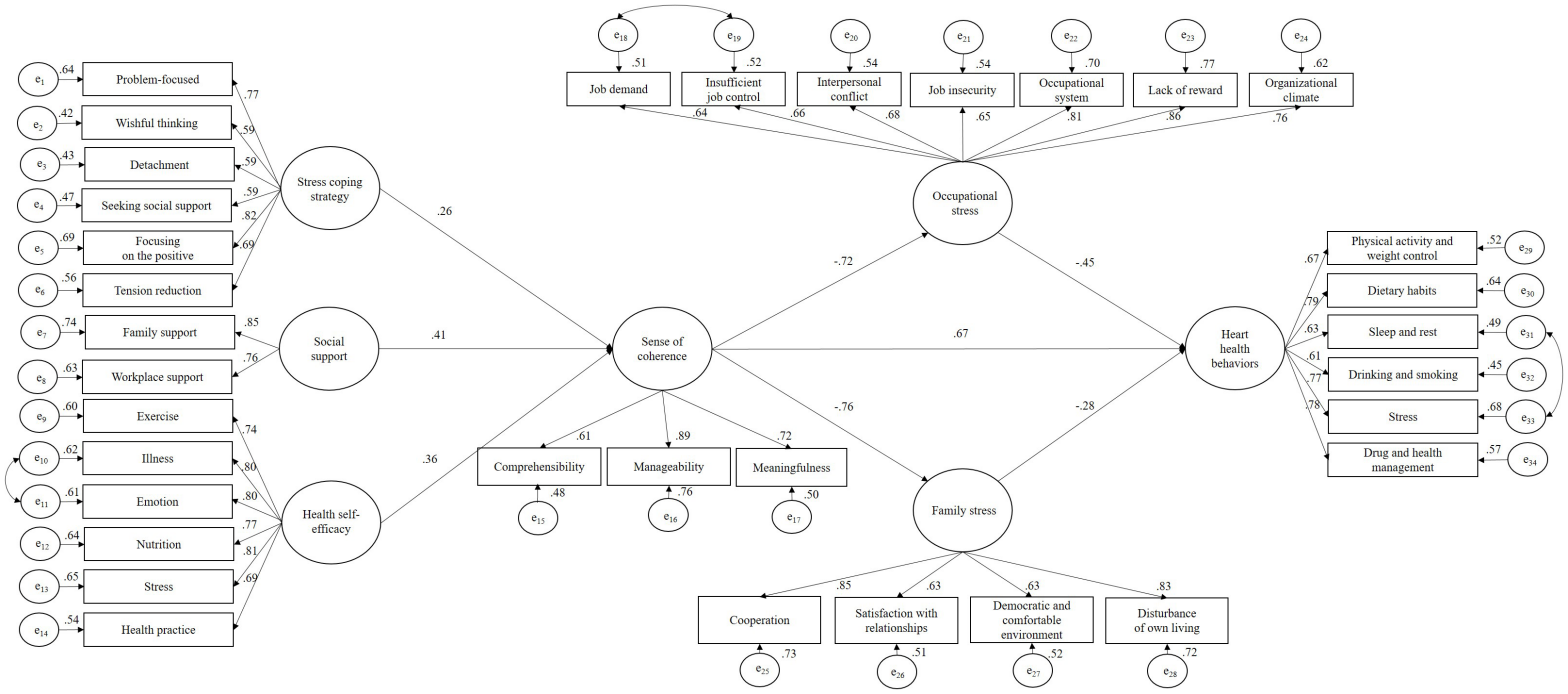
Path diagram of final model.

The initial model’s goodness-of-fit indices were as follows: χ^2^ = 1797.798 (*p* < 0.001), standardized χ^2^ = 2.699, GFI = 0.832, CFI = 0.816, TLI = 0.825, SRMR = 0.064, RMSEA = 0.072 (90% confidence interval [0.068, 0.076]). The model was reviewed comprehensively to improve its fit based on the theoretical rationale by referring to the modification index suggested in AMOS version 23.0. In this study, some observed variables were found to be closely correlated, as described herein: illness (e10) and emotion (e11) in health self-efficacy; job demand (e18) and insufficient job control (e19) in occupational stress; and sleep and rest (e31) and stress (e33) in heart health behaviors. Therefore, some covariance paths between the measurement errors of these variables were added (Figure 2). The modified model’s goodness-of-fit values were acceptable, as follows: χ^2^ = 809.182, standardized χ^2^ = 2.205, GFI = 0.926, CFI = 0.913, TLI = 0.936, SRMR = 0.032, RMSEA =0.061, (90% confidence interval [0.055, 0.066]). All factor loadings were statistically significant (all *p* values <0.001, λ = 0.40–0.85). The correlation coefficients of the latent variables were statistically significant (all *p* values <0.05, |r| = 0.37–0.77). All latent variables showed adequate convergent and discriminant validities (CR = 0.84–0.95, AVE = 0.63–0.83).

### 3.4 Test of SEM: a multiple mediation model

Table 4 shows the multiple mediation model with the two mediators of occupational and family stress. SOC had a significant direct effect on occupational (*β* = −0.72, *p* < 0.001) and family stress (*β* = −0.76, *p* < 0.001). Furthermore, SOC (*β* = 0.67, *p* < 0.001), occupational stress (*β* = −0.46, *p* < 0.001), and family stress (*β* = −0.28, *p* < 0.001) had significant direct effects on heart health behaviors. SOC had significant total indirect effect on heart health behaviors through psychological and environmental factors (*β* = 0.67; *p* < 0.001). Moreover, SOC had a significantly partial mediating effect on heart health behaviors through occupational (*Z* = 3.17, *p* = 0.002) and family stress (*Z* = 2.26, *p* = 0.024; Table 5). Increasing SOC indicated reductions in occupational and family stress; these findings confirm the direct impact of SOC on both occupational and family stress, as well as their collective influence on heart health behaviors.

**Table 4:** Parameter estimates for structural model and standardized direct, indirect, and total effects.

Endogenous variables	Exogenous variables	B	β (SE)	C.R. (*p*)	SMC	Direct effect (*p*)	Indirect effect (*p*)	Total effect (*p*)
SOC	Stress coping strategy	0.62	0.26 (0.15)	4.10 (*p* < 0.001)	0.757	0.26 (*p* < 0.001)	–	0.26 (*p* < 0.001)
	Social support	0.52	0.41 (0.09)	5.68 (*p* < 0.001)		0.41 (*p* < 0.001)	–	0.41 (*p* < 0.001)
	Health self-efficacy	0.55	0.36 (0.11)	5.05 (*p* < 0.001)		0.36 (*p* < 0.001)	–	0.36 (*p* < 0.001)
Occupational stress	SOC	−0.20	−0.72 (0.03)	−6.09 (*p* < 0.001)	0.516	−0.72 (*p* < 0.001)	–	−0.72 (*p* < 0.001)
Family stress	SOC	−0.43	−0.76 (0.04)	−10.72 (*p* < 0.001)	0.581	−0.76 (*p* < 0.001)	–	−0.76 (*p* < 0.001)
Heart health behaviors	SOC	0.61	0.67 (0.09)	6.54 (*p* < 0.001)	0.832	0.67 (*p* < 0.001)	0.54 (*p* < 0.001)	1.21 (*p* < 0.001)
	Occupational stress	−0.73	−0.46 (0.20)	−3.72 (*p* < 0.001)		−0.46 (*p* < 0.001)	–	−0.46 (*p* < 0.001)
	Family stress	−0.22	−0.28 (0.10)	−2.33 (*p* < 0.001)		−0.28 (*p* < 0.001)	–	−0.28 (*p* < 0.001)

β, standardized estimate; C.R., critical ratio; SE, standard error; SMC, squared multiple correlation; SOC, sense of coherence.

**Table 5:** Sobel’s Z test for verifying mediated effect.

Path	Indirect effect (B)	Sobel’s Z	Value of *p*
SOC → Occupational stress → Heart health behaviors	0.15	3.17	0.002
SOC → Family stress → Heart health behaviors	0.09	2.26	0.024

SOC, sense of coherence.

## 4 Discussion

This study used SEM to determine how heart health behaviors were directly and indirectly related to SOC, occupational and family stress, and GRRs (i.e., stress coping strategy, health self-efficacy, and social support) in middle-aged working women based on Salutogenesis. All endogenous variables in this study had direct effects on heart health behaviors in middle-aged working women, and the indirect effects of SOC through occupational and family stress were shown. Thus, the findings of the current study are in line with the Salutogenesis theory, suggesting that life experiences play a role in shaping an individual’s SOC. Moreover, a robust SOC facilitates resource accumulation for coping with stress and effectively navigating tension, thereby impacting an individual’s placement on the well-being spectrum (18, 19).

SOC demonstrated a statistically significant positive association with heart health behaviors in our sample, in alignment with previous findings (24, 48, 49). This suggests that middle-aged working women who can perceive stimuli as structured and predictable, resource availability, and feel that life has a meaning are more likely to adopt heart health behaviors. Particularly, SOC had the greatest influence on heart health behaviors. These result were considered to be meaningful results of verifying the importance of SOC for health, a key factor in Salutogenesis theory (18, 19). It implied that improving heart health behaviors among this population may require first the assessment of SOC, and then the planning of interventions to improve it.

This study also revealed that the relationship between SOC and heart health behaviors was mediated by occupational and family stress. A higher SOC was associated with lower occupational and family stress, and the direct effects of occupational and family stress on heart health behaviors were significant—similar to the direct effect of SOC on heart health behaviors. These findings demonstrated the pivotal role of stress, a key factor in Salutogenesis (18, 19). In other words, even if SOC was low, it was still possible for it to improve heart health behaviors if occupational and family stress were managed.

The significant direct association between SOC and occupational stress found in this study aligns with the results of prior research among Korean dental hygienists (50), reporting that a higher SOC was associated with lower occupational stress. Previous studies have established that occupational stress influences health behaviors, such as alcohol consumption (11), sedentary lifestyle (51), and regular diet (10), and plays a putative role in cardiovascular health, including cardiovascular disease development (15) and increased risk (52). Particularly, in this study, the direct effect of occupational stress on heart health behaviors surpassed that of family stress. Therefore, there is the need to mitigate occupational stress to enhance heart health behaviors in middle-aged working women.

For middle-aged working women, family stress is a significant stressor owing to them often having extensive workloads and familial complexities (16). Our study demonstrated family stress as a pivotal factor influencing heart health behaviors in this population. These findings highlight the need to advocate for more interventions targeting family stress. Additionally, SOC indirectly affected heart health behaviors by lowering family stress in our sample. This aligns with a prior study (23) associating SOC with family-related stress in working women with preschool-aged children. However, the exact role of family stress in heart health behaviors remains a complex issue that is yet to be solved, suggesting the need for further investigation. Still, our finding was similar to that of a previous study (53) showing that low family stress was a predictor of health promotion behavior. Meanwhile, another study (54) analyzed the relationship between family stress and health promotion behaviors for dual earner couples, and reported that the higher the family stress, the higher the health promotion behaviors. The divergence in the findings of the current study and those of the last cited study may be attributed, in part, to the composition of the study sample, where a substantial majority (64.1%) were in their 20s and 30s, and the authors believe that awareness and practice of health behaviors have increased among young workers compared to middle-aged workers.

We incorporated GRRs, namely stress coping strategy, health self-efficacy, and social support, as exogenous variables into the model of this study. Antonovsky’s work (18, 19) framed these resources not as direct influencers of health, but as contributors to the development of SOC. Salutogenesis posits that psychological resources, such as self-efficacy, generate life experiences affecting SOC (55). Aligning with Salutogenesis (19), our study demonstrated that stress coping strategy, health self-efficacy, and social support enhance SOC. This finding resonates with prior empirical research (56–59) that treated these factors as GRRs and revealed their impact on heart health behaviors through SOC. Hence, interventions fostering these resources may help elevate SOC, and subsequently, heart health behaviors in middle-aged working women.

This is the first study to identify factors affecting heart health behaviors in middle-aged working women based on SEM and Salutogenesis. Another strength of the present study is that it shows that SOC can have both indirect (through mediation variables) and direct effects on the heart health behaviors. Furthermore, occupational and family stress were identified as two mediation variables. Our notable findings contribute to a rationale for developing effective strategies and interventions to improve heart health behaviors in middle-aged working women.

Nonetheless, this study had some limitations. First, this study relied on cross-sectional data from middle-aged working women, restricting our ability to establish causality among the variables studied. The findings of this study could be strengthened by using more advanced techniques like bootstrapping to confirm indirect effects. In future research, adopting these methods would enhance the credibility and applicability of the findings. Furthermore, future investigations would benefit from employing longitudinal or randomized trial designs to corroborate the observed effects and confirm causal associations. Second, the data of variables were collected by self-report, but these were directly or indirectly related to the prevalence of the disease. In subsequent study, it is necessary to compare factors affecting heart health behaviors by classifying subgroups according to the presence or absence of disease. Additionally, as the study focused exclusively on Korean women, finding generalizability to middle-aged working women in different geographical regions remains limited. Therefore, future studies should include more diverse participants from other geographical areas to explore the mechanisms influencing heart health behaviors in middle-aged working women.

## 5 Conclusion

This study confirmed that stress coping strategy, health self-efficacy, and social support had significant direct effects on SOC. Furthermore, it found that SOC had a direct effect on occupational and family stress, and that SOC and occupational and family stress had direct effects on heart health behaviors. SOC was also shown to have a significantly partial mediating effect on heart health behaviors through occupational and family stress. Consequently, interventions should focus on effectively fostering SOC, so that middle-aged working women can improve their heart health behaviors. This study’s findings also emphasize that reducing family and occupational stress can improve heart health behaviors in this population.

## Data availability statement

The raw data supporting the conclusions of this article will be made available by the authors, without undue reservation.

## Ethics statement

The studies involving humans were approved by Institutional Review Board of Korea University. The studies were conducted in accordance with the local legislation and institutional requirements. The participants provided their written informed consent to participate in this study.

## Author contributions

JM: Conceptualization, Data curation, Formal analysis, Funding acquisition, Investigation, Methodology, Project administration, Resources, Supervision, Validation, Visualization, Writing – original draft. ES: Writing – original draft, Writing – review & editing.
